# Ultrasound-guided insertion of intra-aortic balloon counterpulsation in intensive care: description of the technique

**DOI:** 10.1186/s13089-020-00166-7

**Published:** 2020-04-21

**Authors:** David Rene Rodriguez Lima, Ever Julián Duran, Ever Leonardo Rojas Díaz, Darío Isaías Pinilla Rojas, Mario Andrés Mercado Díaz, Yury Forlán Bustos Martínez

**Affiliations:** 1 Emergency Medicine and Critical and Intensive Care Medicine, Hospital Universitario Mayor Méderi–Universidad Del Rosario, Bogotá, Colombia; 2 Resident, Critical and Intensive Care Medicine, Hospital Universitario Mayor Méderi –Universidad del Rosario, Bogotá, Colombia; 3 Anesthesiology and Critical and Intensive Care Medicine, Hospital Universitario Mayor Méderi–Universidad del Rosario, Bogotá, Colombia; 4 Critical and Intensive Care Medicine, Hospital Universitario Mayor Méderi–Universidad del Rosario, Bogotá, Colombia; 5 Emergency Medicine Department of Ultrasound and Simulation, Universidad del Rosario, Bogotá, Colombia

**Keywords:** Ultrasound, Intra-aortic balloon counterpulsation (IAoBC), Mechanical circulatory support, Ultrasound education

## Abstract

Intra-aortic balloon counterpulsation (IAoBC) is a mechanical circulatory support device that has been used for more than 50 years, mainly for cardiogenic shock. Although its effect on mortality is controversial, IAoBC is still used in a wide variety of pre- and postoperative clinical settings in cardiac surgery centers. IAoBC has a complication rate of approximately 30%, mostly associated with problems during insertion and malpositioning. Thus, an insertion technique based on the use of ultrasound at the patient’s bedside in the intensive care unit (ICU) is proposed.

## Introduction

Intra-aortic balloon counterpulsation (IAoBC) is used in up to 7% of cardiac surgery patients [1]. IAoBC is used in this context to support coronary circulation and to reduce stress on the left ventricle [2, 3]. Despite the theoretical physiological advantages of IAoBC, its effect on decreasing mortality is controversial. IAoBC has been used in different groups of patients, including in those with cardiogenic shock, regardless of its etiology, refractory angina, acute myocardial infarction, refractory ventricular arrhythmias, mechanical infarct complications (acute mitral regurgitation and ventricular septal rupture), high-risk angioplasty and high-risk cardiac or noncardiac surgery, with different outcomes [1, 4].

The main complications of using IAoBC result from insertion and malpositioning. Rastan et al. [5], in abdominal tomographies, found 68.2% disagreement in balloon length related to the distance between the subclavian artery and the celiac trunk, which was associated with abdominal vessel obstruction and worse clinical outcomes. Vascular lesions can be catastrophic and evident during IAoBC insertion; however, inadvertent dissection of the aorta or iliac artery can go unnoticed, and such dissection is suspected before death in only 20% of cases [6]. Ischemic vascular complications range from 8 to 18%, with critical lower-limb ischemia lower than 1% [7, 8].

Parissis et al. [9] showed that slowed pulse and cold feet were detected in 29.5% of the cases. Ischemia was resolved in most cases by removing the balloon and in 5.8% by thrombectomy; only one patient developed gangrene, which required amputation. Hematoma incidence ranges from 0.4 to 3.9%, and bleeding incidence ranges from 0.4 to 27.7% (mean 5.27 ± 8.5) [10].

IAoBC rupture is not frequent, but can cause a gas embolism and potential entrapment in the arterial tree. This is very rare and occurs in less than 0.5% of cases. The proposed mechanism involves mechanical disruption of the balloon against an atherosclerotic plaque or a calcified aorta and negative pressure created during deflation [11].

The use of ultrasound to perform femoral vascular access during the insertion of the IAoBC [12] and the use of transesophageal echocardiography (TEE) for positioning it [13, 14] has been described; however, since TEE is an invasive procedure, it is not routinely recommended [14, 15]. This article proposes an ultrasound-guided IAoBC insertion technique in the cardiovascular ICU. This technique is original, and similar techniques have not been previously described.

### Technique

When the medical team decides to use IAoBC, the presence of any contraindication for the procedure (aortic valve disease, significant bleeding disorder or infection at the femoral puncture site) should be ruled out, and a clinical evaluation should be performed to rule out severe peripheral artery disease.

After explaining the procedure and asking the patient to sign an informed consent form, the IAoBC is inserted.

**Fig. 1:**
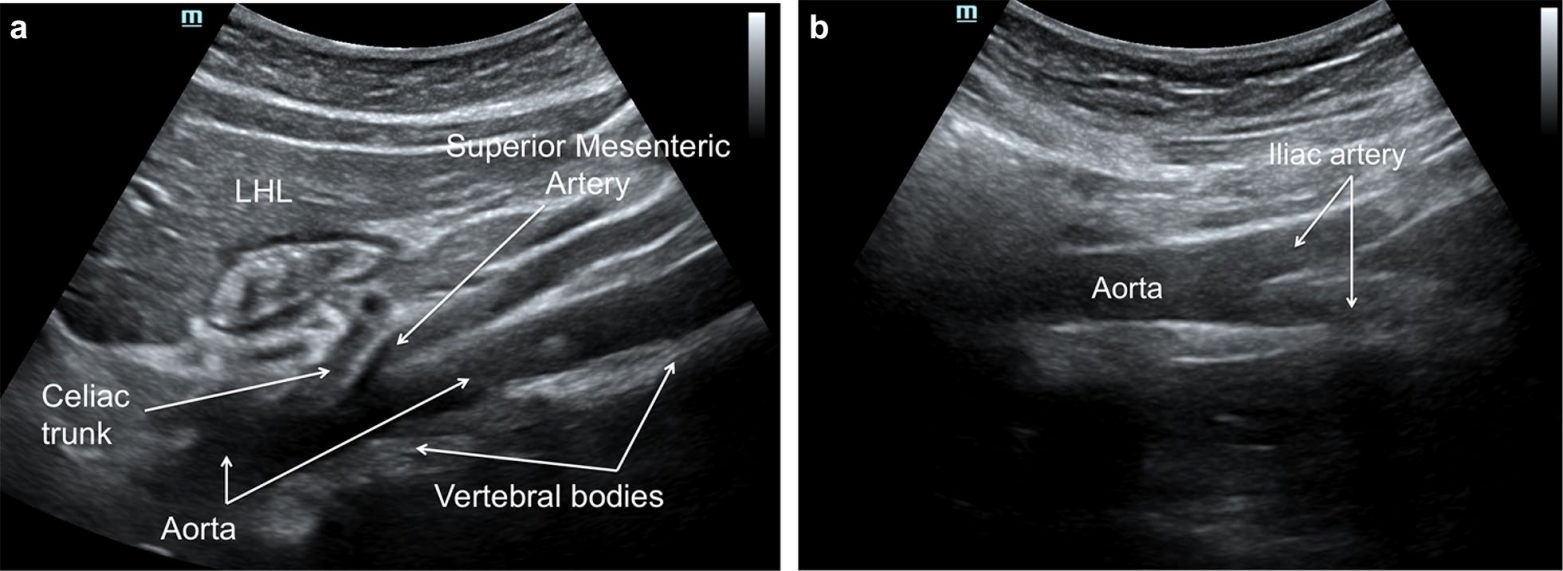
**a** Subxiphoid left paramedian longitudinal section with a low-frequency convex transducer, using the left hepatic lobe (LHL) as a window, showing a pulsatile and anechogenic tubular image, supported on the vertebral bodies, corresponding to the aorta. From the ventral face, the celiac trunk emerges first, and then the superior mesenteric artery runs parallel to the aorta shortly after emerging, following the aorta to the aortic bifurcation. **b** The aorta should be followed distally until the aortic bifurcation

Ultrasound assessment is performed with the patient in the supine position; with a low-frequency (2 to 5 MHz) transducer, in a subxiphoid left paramedian longitudinal section, using the left hepatic lobe as a window, the abdominal aorta is represented as a pulsatile and anechogenic tubular image, supported on the vertebral bodies. From the ventral face, the celiac trunk emerges first, and then the superior mesenteric artery runs parallel to the aorta, following the aorta to the aortic bifurcation. The presence of aneurysms or dissection should not be observed, which are contraindications for the procedure (Fig. 1).

**Fig. 2:**
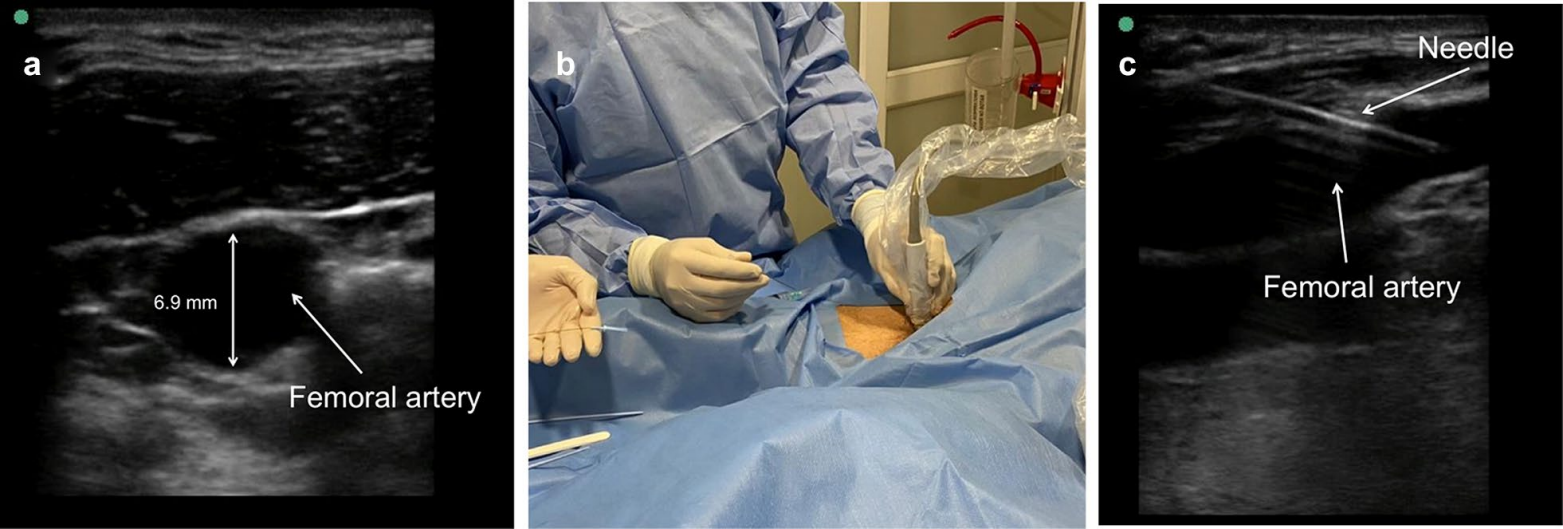
**a** Short-axis visualization of the femoral artery with a high-frequency linear transducer 6.9 mm in diameter. **b** Puncture under direct visualization. **c** Long-axis visualization of the femoral artery as the needle enters the femoral artery during puncture

Then, with a high-frequency linear transducer (6 to 15 MHz), the femoral artery is visualized; the presence of thrombi, aneurysms, atheromas or dissection inside the femoral artery should be ruled out. The diameter of the femoral artery is measured and must be greater than 5 mm (mm) to allow passage of the 8-Fr balloon catheter (2.7 mm). Under sterile conditions, local anesthesia and real-time ultrasound guidance with a high-frequency linear transducer, the common femoral artery is punctured at an angle of 45 degrees or less (Fig. 2).

**Fig. 3:**
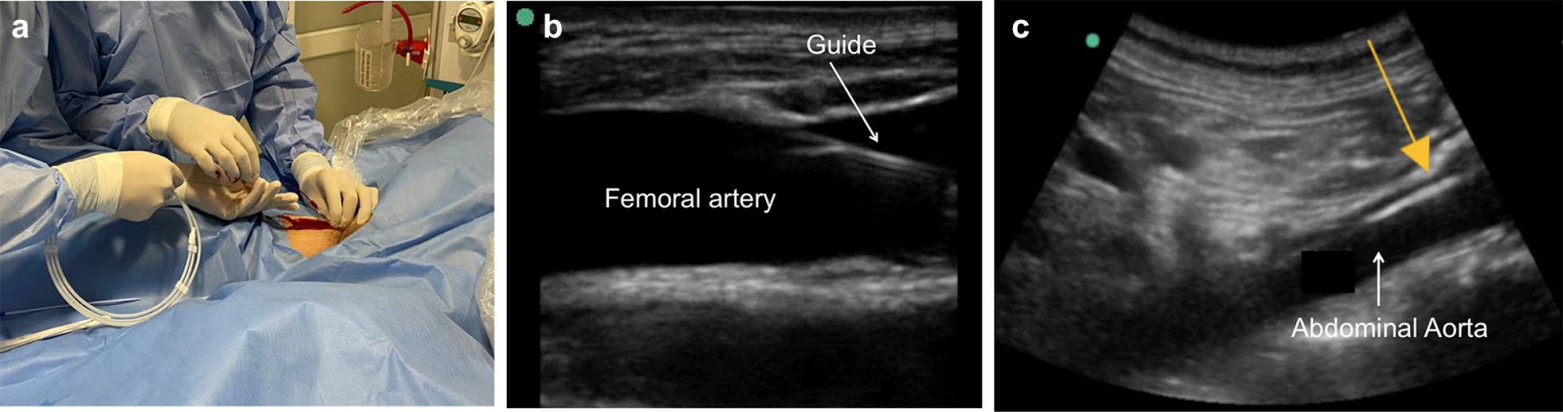
**a** Passage of the metal guide through the femoral arterial puncture. **b** Visualization of the passage of the intravascular guide at the femoral artery with a high-frequency linear transducer. **c** Verification of the metal guide (yellow arrow) in the abdominal aorta with a low-frequency convex transducer

Then, under direct visualization, the metal guide should be advanced without resistance while assessing proper intravascular positioning with the same transducer; the metal guide is inserted 40 to 50 cm, the needle with which the puncture was made is removed, and a second operator with a low-frequency transducer views the left paramedian window again to confirm the presence of the guide in the abdominal aorta (Fig. 3).

After confirming that the guide is adequately positioned, the skin and subcutaneous cellular tissue are dilated, and the 8-Fr catheter is advanced, which is fixed.

**Fig. 4:**
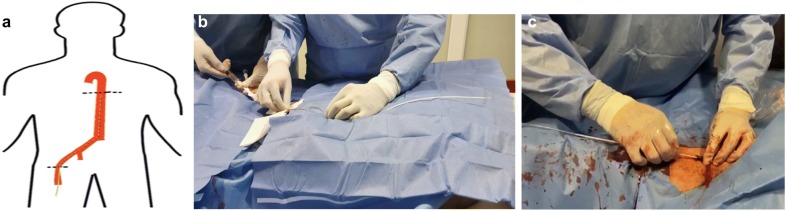
**a** Distance from the second intercostal space to the right common femoral artery. **b** Measurement of the distance for inserting the IAoBC. **c** Passage of the IAoBC

The IAoBC is prepared by creating a vacuum in the helium compartment using a 50-mL syringe and washing the arterial canal with sterile saline. The angle of Louis is measured from the sternum to the navel, and the distance is added to the entry site of the catheter. This distance represents the length of insertion of the IAoBC, which is passed over the metal guide (Fig. 4).

**Fig. 5:**
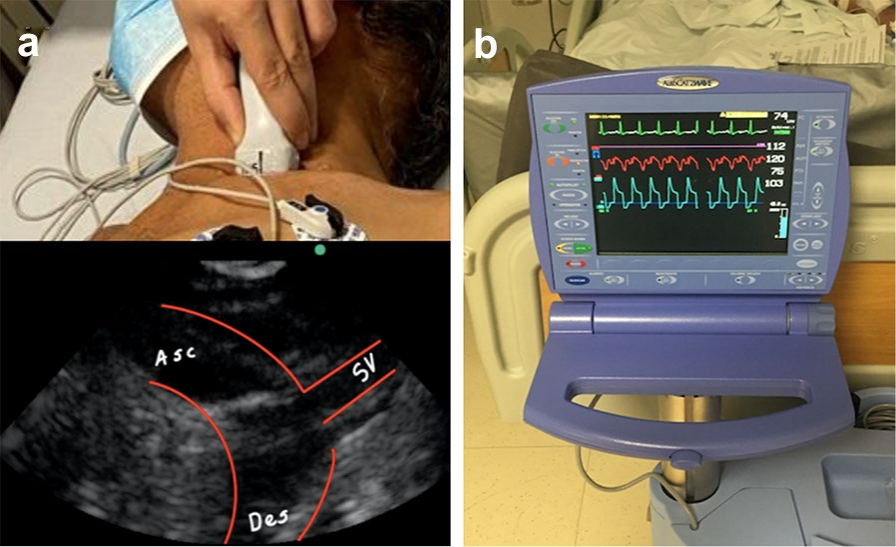
**a** Supraclavicular approach with a phased array transducer, left supraclavicular window, ascending (Asc) and descending (Des) aorta and the left subclavian artery (SV). **b** IAoBC connected to the console and with adequate counterpulsation

After reaching the desired distance of insertion, a left supraclavicular window is created with a phased array transducer, confirming adequate flow and the absence of obstructions of the left subclavian artery by the IAoBC (Fig. 5a), subsequently removing the metal guide and connecting the arterial line to the helium source, starting counterpulsation (Fig. 5b).

**Fig. 6:**
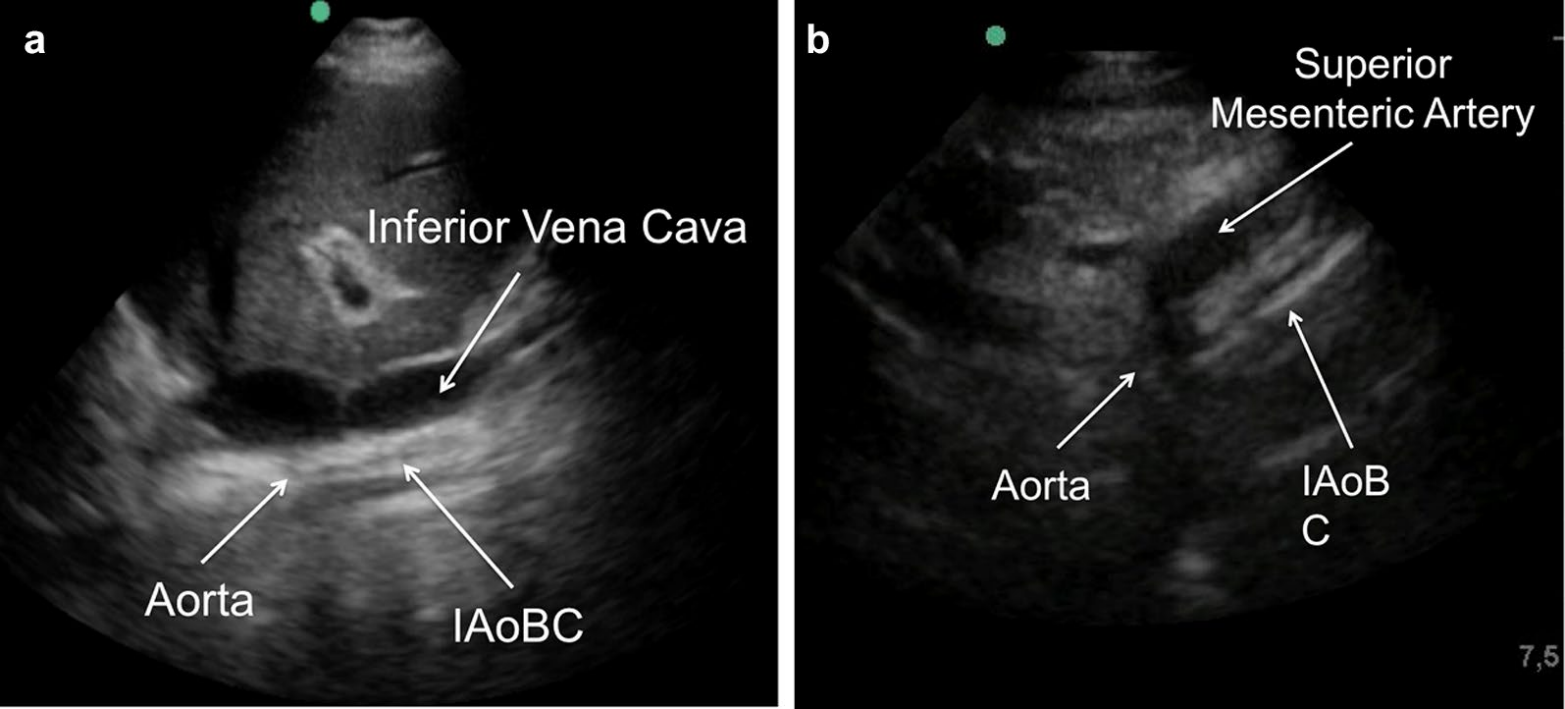
**a** Right, transhepatic, longitudinal window (between the seventh and eighth intercostal space along the axillary midline) with a low-frequency transducer showing the inferior vena cava and the aorta with the functioning IAoBC. **b** Left paramedian longitudinal window to evaluate the exit of the abdominal vessels; in this case, we observe the IAoBC obstructing the superior mesenteric artery exit; thus, the IAoBC was advanced another 5 cm

**Fig. 7:**
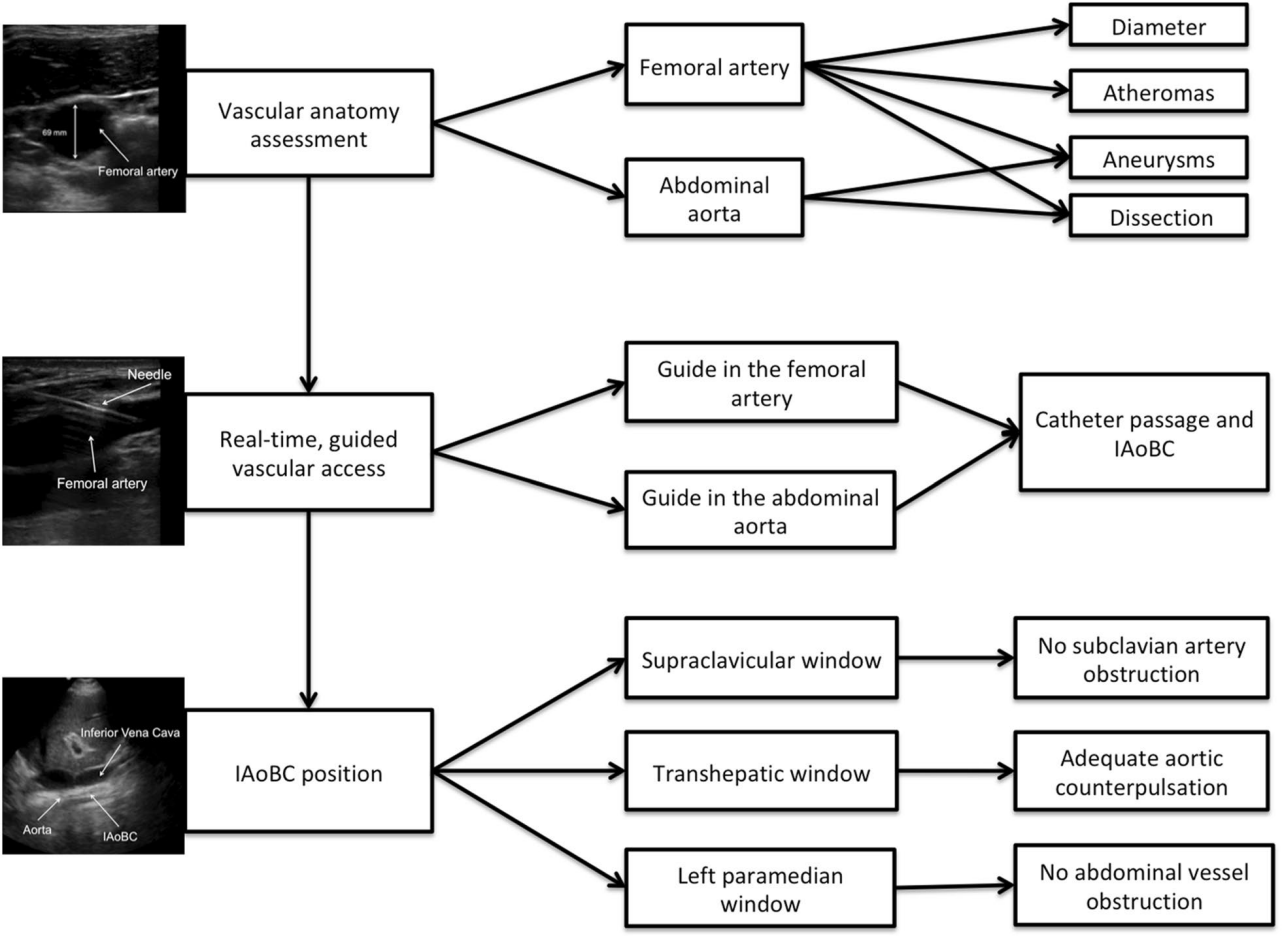
Proposed technique for ultrasound-guided intra-aortic balloon counterpulsation insertion

Finally, adequate counterpulsation with a low-frequency transducer is visualized through a right transhepatic longitudinal window (between the seventh and eighth intercostal space along the axillary midline) (Fig. 6a), imaging again the left paramedian longitudinal section (Fig. 1) to assess the exit of the celiac trunk and upper mesenteric artery, verifying adequate flow and the absence of the IAoBC at this level (Fig. 6b). If all proposed windows and arterial lines can be imaged by ultrasound and adequate diastolic augmentation is confirmed, chest X-ray is not necessary to verify the position of the IAoBC. As a limitation, it remains to be determined whether the use of this technique allows the operator to improve the success rate of the procedure and reduces complications. A summary of the proposed technique for ultrasound-guided intra-aortic balloon counterpulsation insertion is shown (Fig 7).

## Conclusions

An insertion technique based on the use of ultrasound is proposed, allowing safe arterial vascular access, ruling out aortic injuries that can go unnoticed and ensuring adequate positioning. It is expected that its use will reduce the rate of complications associated with the use of IAoBC.

## Data Availability

All data used during this study are available by email at the request of the editorial committee.

## Abbreviations

IAoBC: Intra-aortic balloon counterpulsation
ICU: Intensive care unit
TEE: Transesophageal echocardiogram
